# Integrated Environmental Risk Assessment and Whole-Process Management System in Chemical Industry Parks

**DOI:** 10.3390/ijerph10041609

**Published:** 2013-04-19

**Authors:** Chaofeng Shao, Juan Yang, Xiaogang Tian, Meiting Ju, Lei Huang

**Affiliations:** 1 College of Environmental Science and Engineering, Nankai University, Tianjin 300071, China; E-Mails: ilyc1988@yahoo.com.cn (J.Y.); jumeit@nankai.edu.cn (M.J.); rfxihl@163.com (L.H.); 2 Sichuan Institute of Science and Technology of Environmental Protection, Chengdu 610041, China; E-Mail: txg20032003@yahoo.com.cn

**Keywords:** environmental risk assessment, risk zoning, environmental risk management, chemical industry parks, Tianjin Binhai New Area

## Abstract

Chemical industry parks in China are considered high-risk areas because they present numerous risks that can damage the environment, such as pollution incidents. In order to identify the environmental risks and the principal risk factors in these areas, we have developed a simple physical model of a regional environmental risk field (ERF) using existing dispersal patterns and migration models. The regional ERF zoning was also conducted and a reference value for diagnostic methods was developed to determine risk-acceptable, risk-warning, and risk-mitigation zones, which can provide a risk source layout for chemical industry parks. In accordance with the environmental risk control requirements, this study focused on the three stages of control and management of environmental risk and established an environmental risk management system including risk source identification and assessment, environmental safety planning, early risk warning, emergency management, assessment of environmental effects, and environmental remediation of pollution accidents. By using this model, the environmental risks in Tianjin Binhai New Area, the largest chemical industry park in China, were assessed and the environmental risk zoning map was drawn, which suggested the existence of many unacceptable environmental risks in this area. Thus, relevant suggestions have been proposed from the perspective of the adjustment of risk source layout, intensified management of environmental risk control and so on.

## 1. Introduction

Environmental risk can be thought of as the probability and consequence of the occurrence of environmental pollution incidents caused by spontaneous or anthropogenic activities transmitted through environmental media [1]. Environmental risk can exert harmful or fatal effects on humans and the natural environment [2]. The emergence of studies on environmental risks indicates a strategic turning point for environmental protection because the prediction and management are conducted before pollution accidents arise instead of the “treatment after pollution”. Plus, considerable research has been conducted to satisfy the requirements of environmental risk assessment (ERA) and management.

The identification and control of hazardous sources were initiated by the *Major Hazard Facilities Standard Recommendation* (Advisory Committee of Major-accident Hazards of UK, 1976) and adopted by many institutions in other countries or regions. Frameworks, models, and tools were developed to establish an identification and management system of new gasoline components [3], hazardous substances involved in certain chemical manufacturing processes [4], flammability hazards of hydrocarbon refrigerants [5], transportation and storage of hazardous substances [6,7], and hazardous substances released during flood events [8]. The US EPA and Lakes Environmental Software Company developed a geographic information system (GIS)-based hazardous source information management system to visualize the control of air pollution, water pollution, and health risks.

Quantitative and qualitative methods such as historical statistics, analogy, event-tree analysis, fault-tree analysis, pollutant diffusion model, and fuzzy mathematics have been developed since the 1970s when ERA was conceived [9]. A systematic guidance on ERA projects has been established based on the *Technical Guidelines for Environmental Risk Assessment on Pprojects* in China [10]. For the past two decades, studies have focused on ecological risk assessment in which residential, commercial, and ecologically sensitive areas were considered as receptors and factors [11,12]. Moreover, an integrated approach was proposed to assess health and ecological risks [13].

Acute environmental pollution accidents in the 1970s prompted related institutions and organizations worldwide to investigate the conditions of such accidents and publish guidelines and plans for accident prevention and emergency response. These guidelines include the Organization for Economic Cooperation and Development (OECD) Guiding Principles for Chemical Accident Prevention [14], Awareness and Preparedness for Emergencies at Local Level [15], Hazardous Waste Operations and Emergency Response (US EPA), E2 Plans, and National EPA of Canada. The Seveso accident in 1976 led to the adoption of legislation aimed at the prevention and control of such accidents in the European Union, and *Seveso I* was published to define the major accident hazards of certain industrial activities in 1982 [16]. After many revisions, *Seveso III* has been released with further adaptation of the provisions on major accidents that occurred on 4 July 2012 [17]. The resulting *Seveso* directive now applies to around 10,000 industrial establishments where dangerous substances are used or stored in large quantities, mainly in chemicals, petrochemicals, storage, and metal refining sectors. According to Seveso directive, European countries have developed their respective national accident databases, such as the Major Hazard Incident Data Service of Great Britain [18], Failure and Accidents Technical Information System of The Netherlands [19], Central Major Accident Notification System of Germany [20], and Analysis, Research and Information on Accidents of France [21]. Artificial intelligence and pattern-recognition technologies have recently been used to model oil spill accidents and assess an emergency plan that can support the decision making and the selection of emergency treatment facilities as well as staffing during major oil spill accidents [22].

In China, the frequency of sudden environmental pollution accidents has increased with the increasing social and economic development as well as regional industrialization and urbanization. In the past decade, the whole nation was involved in several sudden environmental accidents [23]. According to a national environmental risk survey conducted by the Ministry of Environmental Protection of China, the environmental safety situation in the country has become serious. The aforementioned survey demonstrated three major characteristics: (1) main risk concern layout; (2) consistent increase in the number of accidents and the vast large areas covered by the accidents; and (3) complexity of the causes for accidents, which adds to the difficulty in early warning and prevention. Meanwhile, secondary environmental incidents caused by natural disasters and safety accidents have been increased [24]. As sudden environmental incidents severely restrain the construction of environment-friendly societies, the principle of “reducing total amount of pollution, improving environmental quality, and preventing environmental risk” was proposed in the 12th Five-Year Plan of the State Environmental Protection of China in December 2011.

The massive expansion of the chemical industry has caused a sudden increase in the number of chemical industry parks. More than 1,200 chemical industry parks have been constructed over the past decade, contributing an output value of more than six hundred billion dollars to China’s industrial economy and approximately 7.5% of China’s gross industrial production. These chemical industry parks mainly manufacture products for the petrochemical and natural gas industry, such as basic organic chemical raw materials, biopharmaceuticals, and new chemical materials. Moreover, chemical industry parks provide storage and transportation of flammable, explosive, toxic, and hazardous chemicals [25]. Potential environmental issues have become more severe, and pollution incidents have occurred frequently because of the large number and various kinds of chemical storage tanks as well as the fragile ecological environment in chemical industry parks that pose considerable risks to human health and the environment [26].

Therefore, enforcing environmental emergency management is the most urgent task for the National Environmental Safety. The key to accomplishing this task is the avoidance of environmental risks based on their characteristics and situations. However, studies focusing on chemical industry park-oriented ERA and emergency management, whole-process environmental management, or complete environmental risk prevention and early-warning system are scarcely reported. In this study, we have comprehensively assessed the scale and intensity of environmental risks in chemical industry parks by building the environmental risk hazard index and vulnerability of receptors based on the simulation of a single environmental pollution accident. In addition, an approach to integrated environmental risk assessment and whole-process management system in chemical industry parks was established on a mesoscale to provide technical support for the development of regional environmental risk management programs.

## 2. Development of Analytical Framework

The quantification of various accidents, hazardous factors, concentration, and ecological risk features of different pollutants is a crucial step in formulating guidelines for a conclusive assessment of a regional environmental risk management program.

### 2.1. Environmental Risk Formulation Mechanism

**Figure 1:**
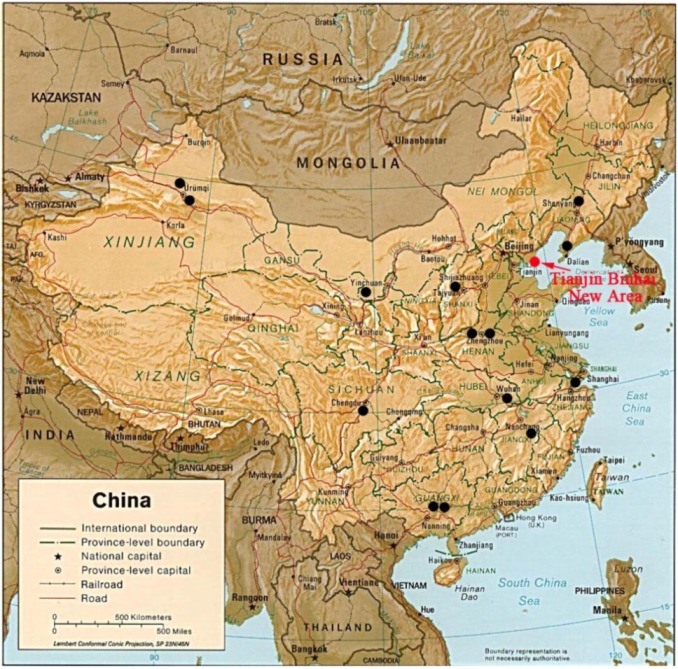
The distribution map of the investigated chemical industry parks.

The primary reason for the frequent accidents in chemical industry parks remains unclear. Thus, this study collected the data from a series of investigations in chemical industry parks. Information on environmental risk sources was also collected from Tianjin Binhai New Area, Urumqi Chemical Industry Park, Ningbo Chemical Industry Park, Guigang Industry Park, and more than 20 other chemical industry parks (see Figure 1). The results were also based on our investigation and management of 15 sudden environmental pollution accidents.

**Figure 2:**
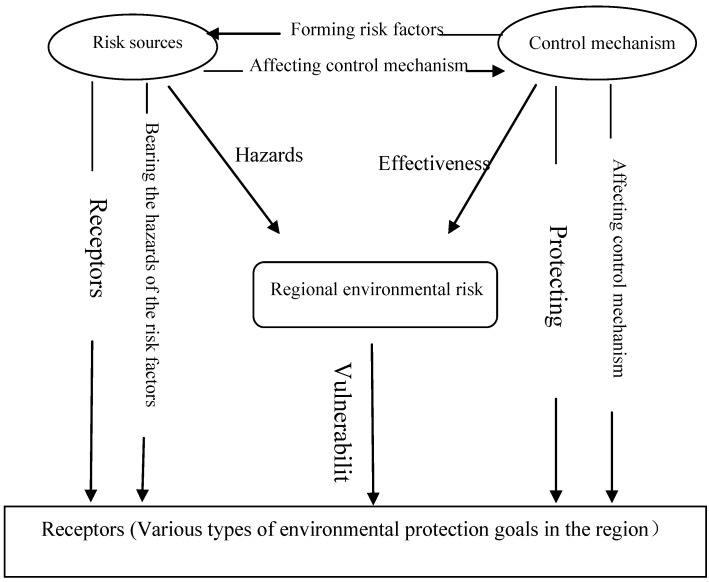
Relationships among the components of the environmental risk system.

The current objective of the ERA is to predict the consequence of a single pollution accident, whereas the domino effect of the accidents and the coupling effect of multiple risks are disregarded for the lack of reliable method. Recently, analyzing multi-hazard risk has become a hot research topic, Kappes and Perles probed the problems and challenges in the analysis of multiple risks [27,28], and Reniers put forward the primary semi-quantitative multi-mode hazmat transport route safety risk estimation methodology which can be use for transportation risk analysis of hazardous substances [6]. As a result of the current ERA, The environmental risk of a single project is acceptable, whereas the regional environmental risk is unacceptable because of multiple risk sources. These risk sources in the same chemical industry parks can affect one another and consequently increase the frequency and severity of accidents. Based on the scene investigations and related statistical analysis, environmental risk sources, control mechanism, and receptors can be considered interactive and interrelated because of the complex environmental risk system with specific structure, functions, and features (see Figure 2).

Figure 2 shows that risk sources, control mechanisms and receptors are the three factors of the environmental risk system. Risk source is the original factor of environmental pollution accidents. Risk source characteristic refers to the levels of toxicity and hazard as well as the amount of substances. Small quantities of substances with high levels of toxicity and hazard can severely harm the environment; similarly, large quantities of substances with low levels of toxicity and hazard can also damage the environment. Control mechanisms will have an effect on the consequence of risk sources. Control mechanisms include the production unit, storage equipment, and other conditions. High-hazard substances with good control have a lower chance of causing environmental accidents compared with poorly secured low-hazard substances. Receptors are various types of environmental protection goals in the region which include residential area with high density and protection zone with ecological function. For the same accident, the more the environmental protection goals are, the more vulnerable the receptors are, and the more severe the hazard consequences are. When the accident happens, the damage will be more fearful if there are more receptors around the accident. Among the three factors, the first factor is the environmental risk of hazardous substances. Sudden environmental pollution incidents become more frequent, and the incident risk becomes more severe when the conditions for the occurrence of an accident are sufficient. The second factor is the vulnerability of the receptor of the environmental risk disaster. The risk increases when there are more receptors.

### 2.2. Research Design

**Figure 3:**
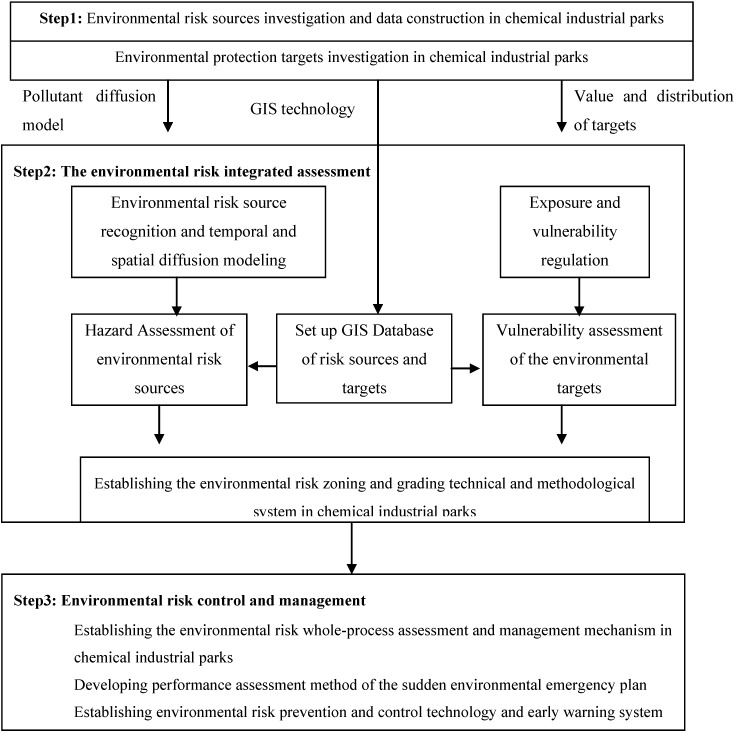
Program design of environmental risk integrated assessment and whole-process management system in chemical industry parks.

This study combined the investigation of risk sources in various chemical industry parks with the ERA of companies in the chemical industry parks to identify the formulation mechanism, the type of environmental pollution incidents inside the parks, and the environmental risk factors. Sudden environmental pollution incidents were evaluated comprehensively and multi-disciplinary theories and methods integrated with modern technical approaches were used systematically. An integrated environmental risk assessment technology was also established based on the severity of risk sources and the vulnerability of the receptors. With reference to the integrated environmental risk assessment, and in combination with the current grading standards of sudden environmental pollution incidents, and technical specifications of risk source recognition, the current study has developed a whole-process mechanism prevention and management of environmental risk for chemical industry parks to support the decision making on environmental safety and management. Figure 3 shows the research flowchart of this study.

## 3. Methodology on ERA and Environmental Risk Zoning

Regional atmospheric environmental risk assessment focuses on middle-level analysis. Based on the study of pollution accidents caused by a single risk source, regional environmental risk assessment method was developed to evaluate the scale and strength of regional environmental risks, and provide technical support for developing management plan for regional environmental risk. Two types of the environmental risk will be discussed in this paper: one is the leakage of harmful substances, and the other is the emission of pollution in accident situation.

### 3.1. Regional ERF Model

Regional ERF is a new spatial distribution pattern formed by various risk factors after the interaction among the single environmental risk incidents [1]. Given that the ERF is composed of *n* particles in the environment, the ERF condition is the same as that of all *n* particles. Likewise, the intensity (*E*) of the ERF is that of all *n* particles. Assuming that *Q_i_* is any one of the particles in ERF and *m* accidents are present in this region, then the intensity at point *Q_i_* can be calculated as follows:

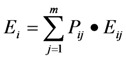
(1)
where *E_i_* is the risk intensity (the space effect of risk source which is irrelevant to risk receptors) of *Q_i_* in the ERF, *P_ij_* represents the probability that the *j*th pollution incident generates the risk field (*E_ij_*) at point *Q_i_*, *E_ij_* denotes the risk field intensity of the pollution incident at point *Q_i_*, and *m* is the number of pollution incidents.

#### 3.1.1. Accident Probability

Generally speaking, more than one risk source exists in a given area. The different accident probability can be obtained from the statistical data. Since 1980s, some international organization began to gather the generic frequencies data for the critical accidents [29,30,31,32]. Accidental Risk Assessment Methodology for Industries (ARAMIS) in the framework of SEVESO directive was considered to be one of the most important achievements, and the probability of accident for deferent risk sources is showed in the ARAMIS final user guide [33]. At present, some accident probability can be detected in *China’s Environmental Statistics Yearbook* and *China Chemical Industry Yearbook* which are published annually.

#### 3.1.2. The Physical Hazard Index

The physical hazard index cannot be directly superimposed in the ERF because the risk properties of different pollutants vary. Therefore, the risk field intensity can be calculated as follows:

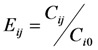
(2)
where *C_j0_* is the environmental threshold of the environmental pollutants in the *j*th pollution incident which can be obtained from *Environmental Quality Standards* in different countries or some Chemicals Database [18,19,20,21], and *C_ij_* is the concentration of the pollution after diffusion. *C_ij_*/*C_i0_*, the environmental hazard index (or simply, “*I*”), is the level of hazard that the pollutants present to the environment.

The risk field generated by any of the risk sources in certain three-dimensional regions can be expressed as a function of *E_i_*:

*E_i_* = *f* (*x_i_*, *y_i_*, *z_i_*, *E_i__j_*) or *E_i_* = *f* (*x_i_*, *y_i_*, *z_i_*, *E_i__j_*, *P_i_*) or *E_i_* = *f* (*x_i_*, *y_i_*, *z_i_*, *I_i_*, *P_i_*) (3)

where *x_i_*, *y_i_*, and *z_i_* are the spatial coordinates of particle Q_i_.

The ERF can then be expressed as follows:

*ERF* = {*E_i_*|*E_i_* = *f* (*x_i_*, *y_i_*, *z_i_*, *I_i_*, *P_i_*)} (4)

### 3.2. Regional ERA Method

Risk value is characterized by the quantity of risk assess, including the probability of occurrence and the degree of hazards of the accident. Risk value can be defined as follows:

*R* = *P* × *C* (5)

where *P* is the probability of the accident which comes from statistical data or empirical data, and the value of *P* is usually 10^−6^–10^−4^ [33]. *C* is the degree of hazard of the accident which is used to indicate the final consequence caused by the accident and counted by means of the number of casualties or economic losses [10,13]. Given the uncertainty of *C* in the regional environmental risk, the consequences of the accidents can be defined by considering the formation process of ERF and its possible consequences:

*C* = *I* × *V* (6)

where *I* is the index of hazard of the accident, and *I* can be determined by *C_ij_*/*C_i0_*. *V* is the vulnerability of the receptors, including the population and environmentally sensitive targets. The vulnerability of the receptors will be scaled according to the related environmental protection laws and regulations in China and the regulation of priority protection for certain targets where there is a sudden pollution incident, as shown in Table 1.

The risk value can then be presented as follows:

*R* = *P* × *I* × *V* = *E* × *V* (7)

Therefore, a new method for risk assessment has been established, in which the risk value is a function related to risk severity and receptor vulnerability.

**Table 1:** Grading for different targets in terms of environmental protection.

Targets for environmental protection		Scale (0–10)
*Natural Reserve*		
	National Natural Reserve	[8,10]
	Natural reserves of province or city	[6,8]
*Basic Farmland*		[4,6]
*Natural Wetland*		[4,6]
*Areas of Dense Population*		
	Density of Population > 3,500/km^2^	[8,10]
	500/km^2^ < Density of Population ≤ 3,500/km^2^	[6,8]
	200/km^2^ < Density of Population ≤ 500/km^2^	[4,6]
	Density of Population ≤200/km^2^	[2,4]
*Unwrought Land Resources*		[0,2]
*Other Types*		[0,2]

### 3.3. Regional Environmental Risk Zoning

Environmental risk zoning can be described as the sequencing of environmental risk levels among regions and the domains of each region. From the mathematical expression of regional environmental risk, the degree of risk caused by sudden environmental pollution incidents depends on the following factors: (1) ERF intensity. Sudden environmental pollution incidents have become more frequent and the risk intensity of pollution incident increases when the condition for the occurrence of accidents becomes more sufficient. (2) Frangibility of the receptor of environmental risk disaster or vulnerability of the ecological environment in the disaster area. The risk increases with more receptors or when the ecological environment becomes more vulnerable.

**Figure 4:**
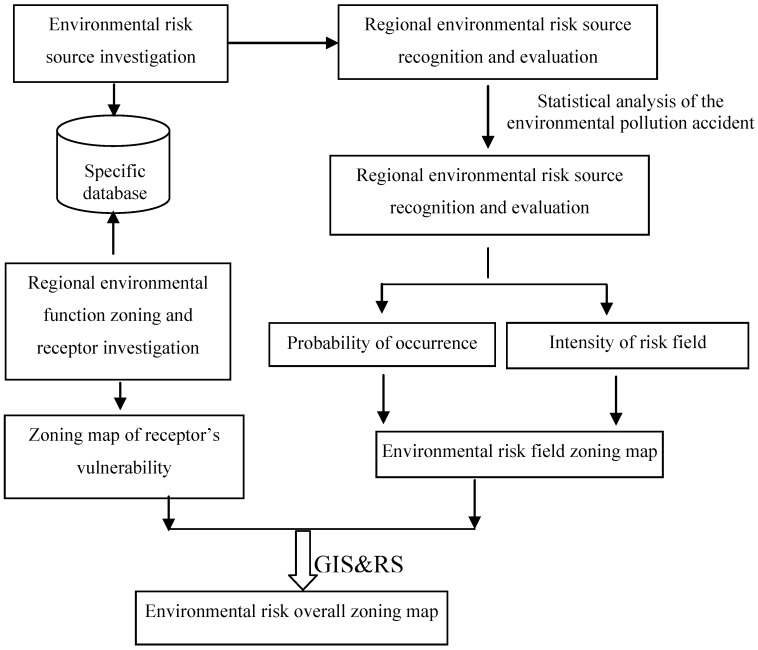
Process flow of environmental risk assessment and zoning in chemical industrial parks.

The assessments of the two aspects above are referred to as hazard and vulnerability assessments. The joint action of these two aspects, which determines the risk level of a sudden environmental pollution incident, is referred to as environmental risk zoning. A zoning map with illustrations reflects the distribution and controlled conditions of sudden environmental pollution incidents. Figure 4 shows the technical flowchart of the map.

The main method of regional environmental risk management and risk zoning is based on the regional discrepancy of risk distribution. According to the results of the regional risk assessment, the vulnerability zoning of receptors and regional environmental risk, combined with the acceptable level of regional environmental risk zones (Table 2), can be classified as follows: (1) risk-acceptable zones with large environmental risk capacity and ability to hold additional risk-developing activities; (2) risk-warning zones saturated with risk capacity and require regional safety layout planning and adjustment to disregard certain capacities for further industrial development; and (3) risk-mitigation zones where the environmental risk is overloaded and the current level should be diminished to an acceptable level by decreasing risk sources and optimizing the layout of risk sources and receptors.

**Table 2:** Regional environmental risk zoning matrix.

Vulnerability of receptors	Degree of hazards of the accidents		
	Low danger areas	Medium danger areas	High danger areas
Low vulnerability areas	Risk-acceptable zones	Risk-acceptable zones	Risk-acceptable zones
Middle vulnerability areas	Risk-acceptable zones	Risk-warning zones	Risk-warning zones
High vulnerability areas	Risk-acceptable zones	Risk-warning zones	Risk-mitigation zones

### 3.4. Case Study

During the past two years, a systematic investigation has been conducted concerning the environmental risk sources and environmental protection targets in Tianjin Binhai New Area, thus it was selected as the case study.

Tianjin Binhai New Area was established in 1994 and it is located in the eastern part of Bohai Sea Region with a surface of 2,270 square kilometers. After twenty years of development, a series of large projects have been established, such as A320 series airliners, a million ton ethane production project, million-ton oil refining project, and the Beijiang Electricity Plant. The total output value of the Tianjin Binhai New Area has reached RMB720.517 billion, thus it has become a new economic engine after Shenzhen Special Economic Zone and Pudong New Area that leads the economic development of the whole region.

**Figure 5:**
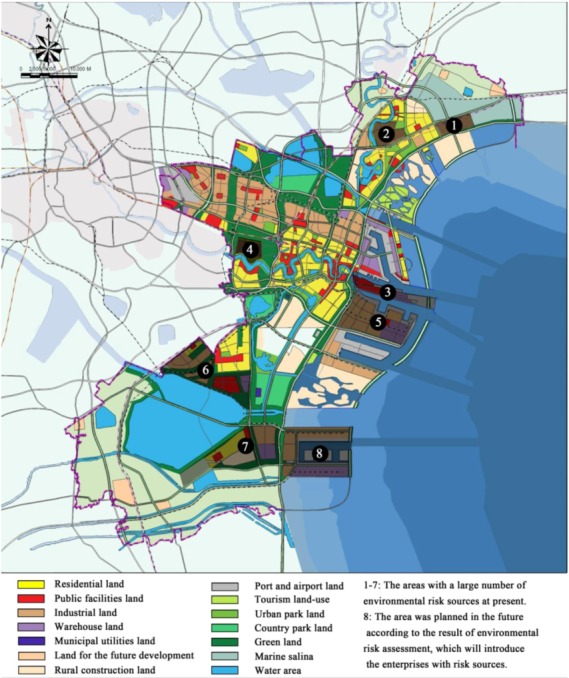
Distribution of the risk sources and the environmental protection goals in Tianjin Binhai New Area.

At present, Tianjin Binhai New Area has formed a complete industrial chain centering on petrochemicals and ranging from oil exploitation to oil refining, ethane and chemical production. And it is regarded as one of the largest chemical regions in the World. According to the survey, there are four major sources of environmental risks in Tianjin Binhai New Area, and distribution of the risk sources was shown in Figure 5.

1. The leakage of toxic and deleterious materials during the production and transportation: focusing on petrochemical industry, marine chemical industry, C1 chemical industry and comprehensive utilization of resources, Tianjin Binhai New Area has carried out several large-scale projects, such as two million-ton oil refining projects, three million tons of ethane production project, and other petrochemical projects concerning million tons of polyvinyl chloride, polyethylene, polystyrene, cacodyl and so on. During the production and transportation, considerable amounts of toxic, deleterious, flammable and explosive materials may leak into the atmosphere, and the spread of benzene, hydrogen sulfide, ammonia, ethane and other materials will harm the ecological environment and people’s health.
2. The storage and transportation accidents of dangerous materials: located in the eastern part of Tianjin Binhai New Area, South Port’s coverage for bulk cargo logistics has reached 12 km^2^. Storage deposits for gasoline and related chemicals have been established and the total storage volume has reached 12.5 million squares. The storage volume of the petrochemicals in the large-scale petrochemical companies is also very high and the demand for transported materials is increasing. Thus, the risk coming from the storage and transportation of the chemical materials is increasing.
3. The oil leakage in the port: according to the incomplete statistics, about 65 leakage accidents took place from 1998 to 2011 in Tianjin Port. Fortunately, oil spill accidents over 10 thousand tons have not happened so far, but with the increasing port handling capacity and increasing numbers of ships passing Tianjin Port, the risk of great oil spill accident is also increasing.
4. The operation of the environmental protection facilities: in Tianjin Binhai New Area, a large-scale petrochemical project group has formed and a large-scale steel smelting base has been established. Due to the large scale of production and operation of the companies, any misoperation of the infrastructure for water treatment, atmosphere pollution treatment and so on will cause considerable pollutants released in short time, which will harm the ecology and human health.

According to *Technical Guidelines for Environmental Risk Assessment on Projects (HJ/T 169-2004)*, the risks of typical environment pollution accidents in Tianjin Binhai New Area were predicted, as shown in Table 3. The results show that the influence range of a single accident will be 200–3,500 m. However, the occurrence of more than one accident will largely expand the influence range, reaching 10 kilometers at the most.

**Figure 6:**
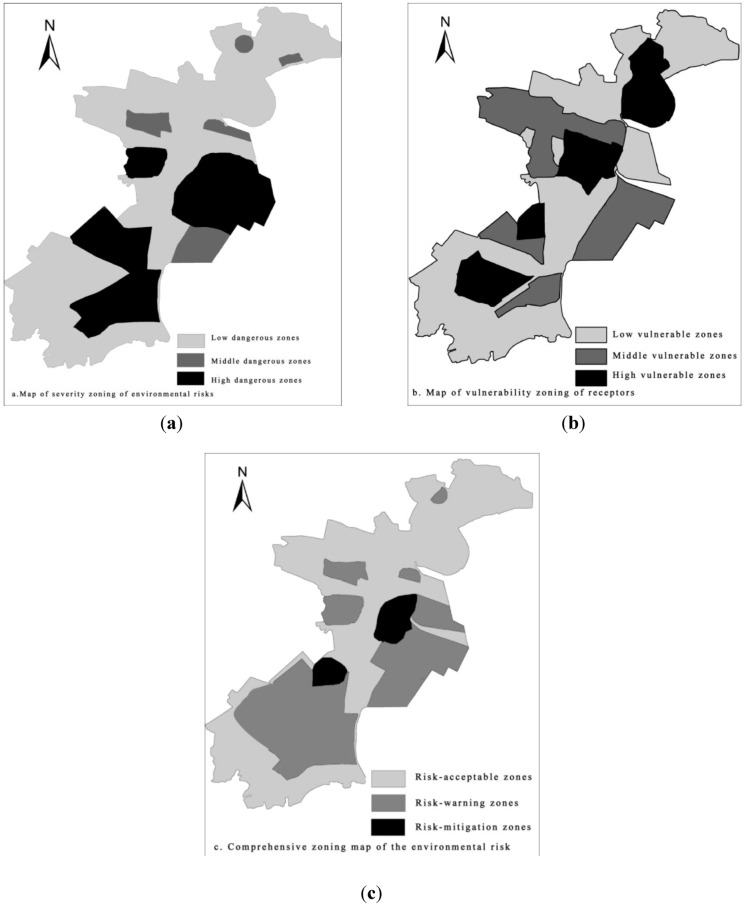
Map of environmental risk zoning in Tianjin Binhai New Area.

Often, the severity of the risk accidents was expressed by the means of the acceptable level of environmental risk [1]. When the value of environmental risk is less than 10^−5^, it belongs to low dangerous areas; when the value of environmental risk is more than 10^−5^, it belongs to high dangerous areas; and when the value of environmental risk is between 10^−5^ and 10^−3^, it belongs to middle dangerous areas. As the results of environmental risk calculation, the environmental risk of Tianjin Binhai New Area can be divided into low-, middle-, and high- dangerous areas, as shown in Figure 6(a).

According to the survey, there are two types of the environmental protection goal in Tianjin Binhai New Area, one type is residence zone, and another is the natural preservation zone. Based on the present situation and development plan of land use, distribution of the environmental protection goals in Tianjin Binhai New Area would be drew as shown in Figure 5. At the same time, vulnerability will be determined according to the scale of the protection targets, and the degree of its vulnerability and value can be calculated by the grading method in Table 1, as shown in Table 4.

Based on the capacity of different environmental protection targets to environmental pollution accidents, we divided the results of vulnerability into three levels. When the value of vulnerability is less than 5, it belongs to the low vulnerability areas; when the value of vulnerability is more than 6, it belongs to the high vulnerability areas; and when the value of vulnerability is between 4 and 6, it belongs to middle vulnerability areas. As the results of vulnerability assessment, Tianjin Binhai New Area will be divided into low-, middle-, and high- vulnerable areas, as shown in Figure 6(b).

**Table 3:** The results of risk calculation of typical environment pollution accidents in Tianjin Binhai New Area.

The main regions with risk sources	Amount of the major risk sources ^1^	Risk transmission paths	Range of influence ^2^	Value of environmental risk
Land area of harbors, which is ③ in Figure 5.	21, which are the oil and chemical storage tanks.	Atmospheric diffusion for the storage and transportation accidents of dangerous materials.	2,000–3,500 me for a single major accident, and 10,500 m for all major accidents.	3 × 10^−4^–5 × 10^−2^
Water area of harbors, which are coastal zones in Tianjin Binhai New Area.	2, which are the two big wharfs.	Water Diffusion for the oil leakages of large ships.	200–1,500 m for an oil leakage, and 8,000 m for all oil leakages.	2 × 10^−5^–6 × 10^−4^
Petrochemical industry areas, which are ②, ⑤, ⑥ and ⑦ in Figure 5.	60, which are the 60 petrochemical enterprises with dangerous chemicals.	Atmospheric diffusion for air pollution accidents, and water Diffusion for water pollution accidents.	500–3,500 m for air pollution accidents, and 200-800 m for water pollution accidents.	5 × 10^−4^–3 × 10^−1^
High emission zones of atmospheric pollutants, which are ① and ④ in Figure 5.	5, which are 5 large-scale enterprises with high use of coal for energy	Atmospheric diffusion for the abnormal discharges of sulfur dioxide and oxynitride.	350–1,200 m for a single enterprise, and the maximum is 2,500 m for all enterprises.	7 × 10^−5^–2 × 10^−2^

Note: ^1^ The major risk sources were recognized according to *Technical guidelines for environmental risk assessment on projects (HJ/T 169-2004)*. ^2^ The influence of environment pollution accidents was calculated based on risk transmission paths of the major risk sources, and diffusion length were determined by the environmental quality standards in China.

Generally speaking, regional environmental risk level is determined by the intensity of sudden pollution accident and the vulnerability of the receptors. As a result, the map of vulnerability zoning of receptors in Tianjin Binhai New Area and the map of severity zoning of environmental risks in Tianjin Binhai New Area can be handle by GIS, thus a comprehensive zoning map of the environmental risk of Tianjin Binhai New Area can be secured by the method in Table 2, as shown in Figure 6(c).

As shown in Figure 6, areas of dark color are risk-mitigation zones which present a high level environmental risk and are beyond the acceptable capacity of the environment. These areas mainly include Dagang District and Tanggu Distict where the population density is comparatively high. In these areas, effective methods of risk supervision must be adopted to reduce the present risk level, which can be achieved by reducing the risk sources or changing the function of these two districts. Reducing the number of the risk sources in these two districts is most effective method after consideration is given to the population density and population number. Areas of light color are risk-acceptable zones where the risk level is relatively low and new industrial development can be conducted. These areas will be appropriate for future petrochemical industry in Tianjin Binhai New Area. However, about 50% of the area belongs to risk-mitigation zones or risk-warming zones which present higher environmental risk. To ensure the regional environmental safety in a better position, a prevention and urgency management system must be established among local residents, enterprises and the government in Tianjin Binhai New Area. What's more, the location of the environmental risk sources should be further optimized to make sure that enough safe distance can be maintained between the environmental protection targets and risk sources.

**Table 4:** The results of vulnerability assessment in Tianjin Binhai New Area.

Category of environmental protection goals	The main regions with environmental protection goals	Value of vulnerability
Natural Reserve	Bei-Da-Gang reservoir, which is the important water area in Figure 5, and belongs to the natural reserves of Tianjin.	8
Natural Wetland	The remaining water areas in Figure 5.	5
Basic Farmland	The rural construction lands in Figure 5.	5
Areas of Dense Population	Dagang residential zone, Tanggu residential zone and Hangu residential zone with the population density of 2,100–5,000 per square km, which are the residential lands in Figure 5.	7–9
	The industrial concentration district the population density of 300–1,500 per square km, which the industrial lands in Figure 5.	5–6
Other Types	The remaining land utilization in Figure 5.	0–2

## 4. Theoretical Framework of Whole-Process Management Mechanism of Environmental Risk

**Figure 7:**
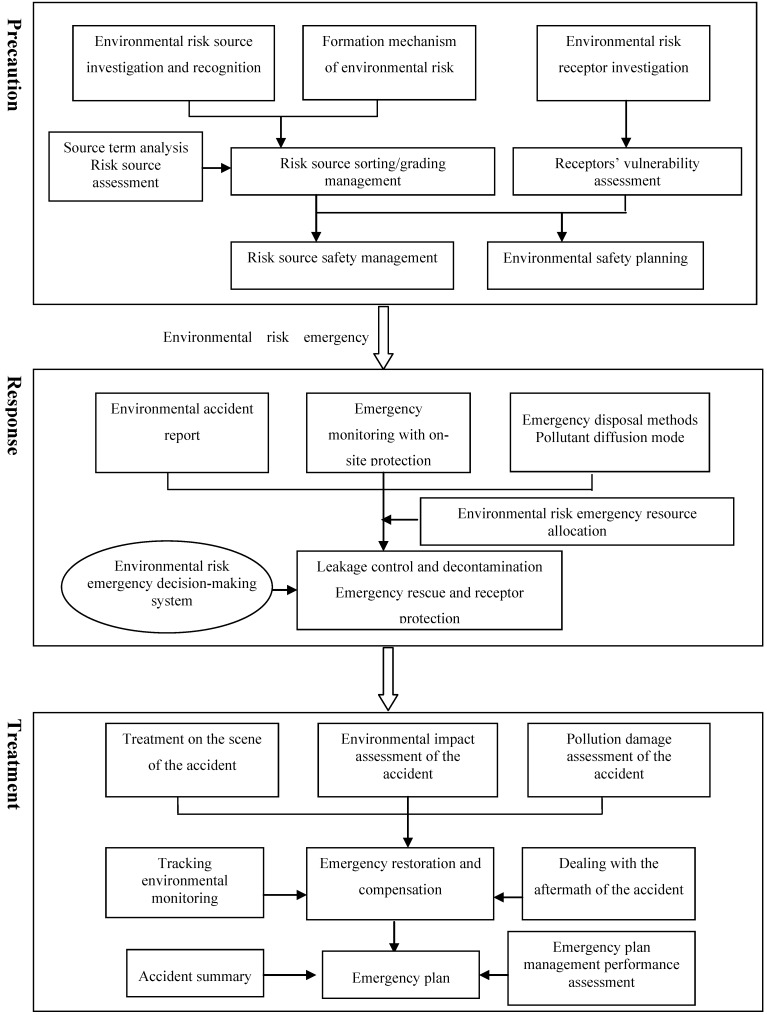
Framework of environmental risk whole-process management mechanism.

On the basis of the formation mechanism of the regional risk and the ERA results, the whole-process management mechanism of environmental risk adopts regional risk zoning as well as the optimization and adjustment of the risk source layout. The mechanism also focuses on the early-warning management of risk source and emergency control to address every aspect of environmental risk accidents. These accidents involve three factors: environmental risk sources, control and management mechanism, and environmental risk receptors. Figure 7 shows the flowchart of the whole-process management mechanism of environmental risk.

Precaution emphasizes the control and management of environmental risk sources. Each potential risk source of pollution incidents should be controlled. The relationship between environmental risk sources and receptors should be coordinated from the origin, and regional environmental safety planning should be implemented. Response to incidents as they transpire emphasizes timely and effective treatment of pollution incidents after the release of risk factors and the launch of an environmental risk emergency plan to minimize the possible effect of pollution incidents. Post-incident treatment focuses on environmental restoration measures in accordance with the effect of the incidents as determined by environmental risk real-time assessment to revise and improve the environmental risk emergency plan system. Therefore, the whole-process management of environmental risk includes four aspects: environmental risk source management, environmental safety planning, environmental risk emergency management, as well as environmental restoration and ecological compensation.

### 4.1. Environmental Risk Source Management

Environmental risk source management is the foundation of environmental risk management and provides means for its transformation from a positive and lagging state to a whole-process state. An effective environmental risk source management can be established by investigating the environmental risk sources, environmental risk source assessment, and environmental risk source grading and classification system.

1. Investigation and recognition of environmental risk sources. Investigation of environmental risk sources should be promoted and basic supervision of the environmental risk and chemicals should be enhanced with focus on important industries such as the chemical and petrochemical industries to establish an effective management mechanism for environmental risk prevention and chemical supervision policies. In addition, the investigation of environmental risk sources should take into consideration the formulation mechanism of the main pollutants during urbanization and the use of quantitative evaluation system; the regional pollutants can be sorted and classified according to risk. The investigation should also consider the basic parameters that establish toxicity, exposure level, and environmental chemical properties as evaluation indices. The purpose is to obtain quantitative evaluation results of regional hormone pollutants sorted and classified according to risk, as well as to identify the pollutants that should be controlled in surface water, air, and soil environments in a typical industrial concentration area.
2. Environmental risk source assessment. Based on the analysis of safety system project and causation theory of pollution incidents, the corresponding relationship between the hazard level of environmental risk sources and environmental pollution incidents can be established by using current identification methods of major environmental risk sources. These methods consider the possible consequences of environmental risk sources and the distribution of environmentally sensitive targets in the surrounding environment. To achieve a dynamic management of environmental risk sources, standards for grading and classifying environmental risk sources should be established. Different types of sudden environmental pollution incidents can then be categorized according to fixed sources (storage tank, storage and production of fuels, raw materials, medicines, and intermediates), mobile sources (vehicles, ships, and aircrafts for transporting dangerous goods), pipelines (oil and gas), and other indices such as the degree of harm, frequency of occurrence, and characteristics of pollutants.
3. Environmental risk source management system. An environmental risk source management system is the solid foundation of environmental risk source management. This system is essential to whole-process management of environmental risk for compliance with related laws, regulations, and standards. The Emergency Response Law of the PRC failed to direct clearly the environmental risk precaution and management because of deficiencies in implementing rules and supporting regulations. Thus, effective environmental risk precaution and management mechanism as well as chemical supervision policies and measures should be established by standardizing emergency plans for sudden environmental pollution incidents in key areas (industrial zones) and enterprises. Environmental risk sources at different levels should be distinguished to improve management efficiency. For the risk sources with high hazard which can cause environmental pollution and ecological damage, the strict management system needs to be carried out.

### 4.2. Regional Environmental Safety Planning

To minimize regional risk and protect human beings and environmentally sensitive targets, the regional environmental safety planning optimizes the environmental risk source layout within the region, coordinates the relationship between risk sources and receptors, establishes an emergency resource allocation system, and reduces risk in key industrial areas.

1. Environmental risk source layout. Environmental risk source dispersal is a method for reducing the overall regional environmental risk by decomposing a specific risk source into smaller risk sources. Risk adjustment and control are applied to major environmental risk sources that cannot meet the safety planning objectives in the region to increase regional safety. However, new enterprises with risk sources are selected according to the distribution of environmentally sensitive targets, environmental background, and other factors at the regional level.
2. Protection of receptors. To protect the population and environmentally sensitive targets, safety distances are investigated and verified for different industrial activities or facilities, sites and residential buildings, public regions, and other important regions by considering the regional development orientation, properties, scale, and state of spatial development. The safety distance depends on the type of industrial activity as well as the type and level of hazard. With risk management, the vulnerability of the receptors can be decreased with increased resilience.
3. Allocation of emergency resources. The rational use of resources is essential to the implementation of an environmental pollution emergency plan. However, shortage and irrational allocation of emergency resources are key constraints in the current emergency management of sudden environmental pollution incidents in China. The establishment of an effective emergency resource allocation system is an important measure of receptor safety. This system is based on the type of environmental risk incidents in industrial areas and the distribution of environmental risk sources and receptors. Moreover, the emergency material reserve protection system as well as the regulatory, production, reservation, allocation, and emergency distribution systems of important emergency supplies should be gradually perfected.

### 4.3. Environmental Risk Emergency Management

Environmental risk emergency management should be implemented to ensure steady and safe operation of chemical industry parks. It includes the following tasks: ensuring a prompt and effective post-incident treatment and protection of the receptors of environmental risk, establishing an emergency planning system and decision making, and creating an emergency response system for sudden environmental pollution incidents.

1. Emergency planning system. An effective emergency plan directs an efficient and systematic emergency operation, and ensures prompt action on risk sources at different levels, and different distributions, of the receptors. There are five aspects to consider in emergency planning as follows: emergency organization; management and command system; emergency engineering and rescue system; support system with synthesized coordination, prompt response, and fully prepared safety and supply systems; and integrated rescue abilities of the emergency team.
2. Emergency decisions. An emergency management system with classified management and graded responsibility should be established and perfected. A computer-based emergency decision support system that can assist decision makers in semi-structured or unstructured interactive decision making can be established. Information technology, management science theory, computer science, and other disciplines related to pollutant leakage control and pollution cleanup, emergency rescue and receptor protection, and communication on risk information can be used to develop this system. An emergency management system includes the following key steps: development of a technology to control and manage environmental risk sources; risk prevention through knowledge of emergency treatment technologies that counter the effects of hazardous substances; preventions; establishment of an emergency monitoring network of risk sources; and use of emergency treatment technologies such as rapid-closure for pollutants and rapid reduction of upper-air diffusion of pollutants. In addition, access to official channels should be provided for awareness on the progress, cause, and results of emergency management measures.

### 4.4. Assessment and Environmental Restoration after the Occurrence of Environmental Pollution Incidents

Post-incident treatment, which resolves the social and psychological trauma as well as the unseen effects of environmental pollution, alleviates the damage caused by environmental pollution and initializes environmental restoration. Restoration is the last phase in the whole-process management of environmental risk. The common situation in which emergency treatment is emphasized and environmental restoration is disregarded always increases the subsequent effects of environmental pollution, thus aggravating human health and the ecological system.

1. Assessment after environmental pollution incidents. This process includes the following: (a) evaluation of the effects exerted by major pollution incidents on the environment and introduction of compensation schemes; (b) prediction of the medium- and long-term effects of pollution incidents as well as proposal of corresponding pollution-mitigation measures and environmental protection programs; (c) identification of appropriate measures, namely, early warning, response, rescue operations, and pollution control; (d) improvement of environmental emergency plans according to the emergency response situation; and (e) investigation of the causes for pollution incidents to provide evidence for responsibility confirmation of other pollution incidents, treatments, and reference for the prevention of environmental risks.
2. Environmental restoration. Environmental restoration and ecological compensation schemes should be developed in response to the possible medium- and long-term effects of pollution incidents on the ecosystems (e.g., proposing ecological compensation programs for affected farmland to protect the safety of agricultural products and introducing restoration programs for the affected environment to ensure that environmental quality standards are attained).

## 5. Conclusions

Unlike a single construction project, regional ERA and management are classified as new strategies in environmental risk management. These strategies resolve the multiple uncertainties of risk sources and their effects, non-additivity of the consequences of accidents, and other factors. In this study, a systematic analysis of the mechanisms underlying environmental risks was conducted, and an ERA method based on risk field theory was established. Theories and methods were proposed for environmental risk zoning in industrial parks.

1. A pollutant hazard index analysis and assessment methods were proposed based on the intensity and hazard of pollutant leakage. A basic physical model and the characterization techniques of regional ERF were established by using related predictive modeling. Based on these, we proposed that the consequences of environmental risks caused by accidents consist of two parts: the intensity of atmospheric ERF (the product of the probability of accident occurrence and the risk index of pollutants) and the vulnerability of the receptors.
2. A risk load theory-based path was designed to optimize the regional environmental risk source layout as well as to protect the population and major sensitive targets of the environment against the risk source layout in industrial parks.
3. The mechanism for whole-process environmental risk management was established. This mechanism includes a set of risk management strategies such as major pollution accident prevention, sudden emergency capacity building, and environmental pollution accident compensation. The three stages of environmental risk accidents covered prevention, response during accidents, and disposal after accidents.

The theories and methods of regional ERA and management remain in the development stage. In particular, the regional environmental risk spatial and temporal zoning, multi-risk coupling and risk-field superimposition as well as regional environmental risk capacity allocation are in the exploratory stage. Despite the establishment of the ERA technology and management mechanism in chemical industry parks, further tests, optimization, and upgrade, combined with application practices, are required, which should be based on the investigation and evaluation of risk sources in multiple chemical industry parks.

## References

[1] Bi J., Yang L., Li Q.L. (2006). Regional Environmental Risk Analysis and Management.

[2] Roselló M.J., Martinez J.M., Navarro B.A. (2009). Vulnerability of human environment to risk: Case of groundwater contamination risk. Environ. Inter..

[3] Huttunen H., Wyness L.E., Kalliokoski P. (1997). Identification of environmental hazards of gasoline oxygenate tert-amyl methyl ether (TAME). Chemosphere.

[4] Manassaram D.M., Orr M.F., Kaye W.E. (2003). Hazardous substances events associated with the manufacturing of chemicals and allied products. J. Hazard. Mater..

[5] Colbournea D., Suenb K.O. (2004). Appraising the flammability hazards of hydrocarbon refrigerants using quantitative risk assessment model. Int. J. Refrig..

[6] Reniers G.L.L., de Jongh K., Gorrens B. (2010). Transportation Risk Analysis tool for hazardous Substances (TRANS—A user-friendly, semi-quantitative multi-mode hazmat transport route safety risk estimation methodology for Flanders. Transport Res. Transport Environ..

[7] Bernechea E.J., Viger J.A. (2013). Design optimization of hazardous substance storage facilities to minimize project risk. Safety Sci..

[8] Landucci G., Antonioni G., Tugnoli A., Cozzani V. (2012). Release of hazardous substances in flood events: Damage model for atmospheric storage tanks. Reliab. Eng. Syst. Safe..

[9] Li Q., Bi J., Yang J. (2005). Fuzzy evaluation model for industry park’s environmental risk management performance and its apply. Environ. Protect..

[10] Environmental Protection Apartment of China Technical Guidelines for Environmental Risk Assessment on Projects (HJ/T 169-2004). http://kjs.mep.gov.cn/hjbhbz/bzwb/other/pjjsdz/200412/t20041211_63369.htm.

[11] Pollard S., Purchase D., Herbert S. A Practical Guide to Environmental Risk Assessment for Waste Management Facilities (Guidance Note 25 Version 2). http://infohouse.p2ric.org/ref/16/15252.pdf.

[12] Venter H.S., Eloff J.H.P., Li Y.L. (2008). Standardizing vulnerability categories. Comput. Secur..

[13] Suter G.W., Vermeire T., Munns W.R., Sekizawa J. (2005). An integrated framework for health and ecological risk assessment. Toxicol. Appl. Pharm..

[14] (2003). Guiding Principles for Chemical Accident Prevention, Preparedness and Response.

[15] UNEP APELL-Information and Tools for Emergency Response Planning, 2003. www.unep.fr/scp/sp/programme/.

[16] (1990). COUNCIL DIRECTIVE 82/501/EEC on the Major Accident Hazards of Certain Industrial Activities.

[17] European Communities Directive 2012/18/EU of the European Parliament and of the Council. http://eur-lex.europa.eu/LexUriServ/LexUriServ.do?uri=CELEX:32012L0018:EN:NOT.

[18] AEA Technology plc (2010). Major Hazard Incident Data Service.

[19] Netherlands Organization for Applied Scientific Research Failure and Accidents Technical Information System. www.factsonlin.nl/.

[20] Federal Envimnmentel Agency Central Major Accident Notification System. www.infosis.uba.de/index.php/en/site/13947/zema/index.html/.

[21] French Ministry of Ecology, Energy, Sustainable Development Analysis, Research and Information on Accidents. www.aria.development-durable.gouv.fr/.

[22] Mago V.K., Bhatia N. (2012). Cross-Disciplinary Applications of Artificial Intelligence and Pattern Recognition: Advancing Technologies.

[23] National Bureau of Statistics of China, Environmental Protection Apartment of China (2011). China Environment Statistical Yearbook.

[24] The editorial department of environmental protection (2011). Preventing environmental risks and ensuring environmental safety. Environ. Protect..

[25] Meel A., O’Neill L.M., Levin J.H., Oktem U., Keren N. (2007). Operational risk assessment of chemical industries by exploiting accident databases. J. Loss Prevent. Proc..

[26] Shi C., Duo Y.Q. (2010). Safety Capacity for hazardous chemicals transportation of chemical industry park. Transport Standard..

[27] Kappes M.S., Keiler M., Elverfeldt K.V., Glade T. (2012). Challenges of analyzing multi-hazard risk: A review. Nat. Hazards..

[28] Perles Roselló M.J., Prados F.C. (2010). Problems and challenges in analyzing multiple territorial risks: Methodological proposals for the development of multi-hazard mapping. De la AGE y Otros..

[29] Crossthwaite P.J., Fitzpatrick R.D., Hurst N.W. (1988). Risk assessment for the siting of developments near liquefied petroleum gas installations. IChemE Symp. Ser..

[30] COVO Commission (1981). Risk Analysis of Six Potentially Hazardous Industrial Objects in the Rijnmond Area, a Pilot Study: A Report to the Rijnmond Public Authority.

[31] Logtenberg M.T. (1998). Derivation of Failure Frequencies for LOC Cases.

[32] Spouge J. (2005). New generic leak frequencies for process equipment. Proc. Safety Prog..

[33] The Major Accident Hazards Bureau, European Commission Accidental Risk Assessment Methodology for Industries in the Framework of SEVESO II Directive. http://mahb.jrc.it/index.php?id=440.

